# Investigating the effects of inter-annual weather variation (1968–2016) on the functional response of cereal grain yield to applied nitrogen, using data from the Rothamsted Long-Term Experiments

**DOI:** 10.1016/j.agrformet.2019.107898

**Published:** 2020-04-15

**Authors:** John W.G. Addy, Richard H. Ellis, Andy J. Macdonald, Mikhail A. Semenov, Andrew Mead

**Affiliations:** a Computational and Analytical Sciences Department, Rothamsted Research, UK; b School of Agriculture, Policy and Development, University of Reading, UK; c Sustainable Agriculture Sciences Department, Rothamsted Research, UK; d Plant Science Department, Rothamsted Research, UK

**Keywords:** Crop yield, Wheat (*Triticum aestivum* L.), Barley (*Hordeum vulgare* L.), Nitrogen, Rainfall, Temperature, Climate change

## Abstract

The effect of weather on inter-annual variation in the crop yield response to nitrogen (N) fertilizer for winter wheat (*Triticum aestivvum L.*) and spring barley (*Hordeum vulgare L.*) was investigated using yield data from the Broadbalk Wheat and Hoosfield Spring Barley long-term experiments at Rothamsted Research. Grain yields of crops from 1968 to 2016 were modelled as a function of N rates using a linear-plus-exponential (LEXP) function. The extent to which inter-annual variation in the parameters of these responses was explained by variations in weather (monthly summarized temperatures and rainfall), and by changes in the cultivar grown, was assessed.

The inter-annual variability in rainfall and underlying temperature influenced the crop N response and hence grain yields in both crops. Asymptotic yields in wheat were particularly sensitive to mean temperature in November, April and May, and to total rainfall in October, February and June. In spring barley asymptotic yields were sensitive to mean temperature in February and June, and to total rainfall in April to July inclusive and September.

The method presented here explores the separation of agronomic and environmental (weather) influences on crop yield over time. Fitting N response curves across multiple treatments can support an informative analysis of the influence of weather variation on the yield variability. Whilst there are issues of the confounding and collinearity of explanatory variables within such models, and that other factors also influence yields over time, our study confirms the considerable impact of weather variables at certain times of the year. This emphasizes the importance of including weather temporal variation when evaluating the impacts of climate change on crops.

## Highlights

• Variation in yield-nitrogen responses explored using long-term experiments (LTEs).
• Yield-nitrogen relationship modelled using linear-plus-exponential function.
• Inter-annual variation in parameters explained using monthly weather summaries.
• Modelling allows separation of effects of environmental and agronomic factors.
• Selected monthly weather variables associated with key crop development processes.

## 1 Introduction

Many factors affect cereal crop yields including weather and climate, soil structure and fertility, pest, weed and disease incidence, previous cropping, cultivar, fertilizer applications and other agronomic practices. It is predicted that to keep pace with rising food demand, global crop production will need to be 60% greater than current levels by 2050, with fewer inputs and no increase in agricultural land use (FAO, 2017). This intensification of crop production must also accommodate adaptation to global change in climate: average global temperature in 2016 was 1.43°C above the 20th century average (NOAA, 2017); and warming is anticipated to continue throughout the remainder of this century, including more frequent high temperature extremes and more variable rainfall, due to anthropogenic emissions of greenhouse gases (IPCC, 2014). Variations in rainfall have long been associated with variations in the grain yield of wheat (Fisher, 1925; Lawes and Gilbert, 1871) and spring barley (Wishart and Mackenzie, 1930). These variations in rainfall may contribute to an increased frequency of drought conditions, reducing plant growth (da Silva and Kay, 1997). Whilst the crop response to drought develops over a comparatively long-time scale (Peña-Gallardo et al., 2019), even brief exposure to high temperature at sensitive stages of crop development, such as anthesis, can reduce wheat grain yield considerably, largely due to lower seed set (Ferris et al., 1998; Wheeler et al., 1996). This has also been observed in other crops (see Wheeler et al., 2000). Understanding both the short-term impacts of weather and the long-term impacts of climate on crop production is essential to future food security in a changing climate.

The Rothamsted Long-Term Experiments are some of the oldest continuous studies of crop production in the world. In particular, the Broadbalk Wheat Experiment was established in 1843 (Lawes, 1847) and is recognised as the oldest continuing scientific experiment in the world (www.guinnessworldrecords.com/world-records/longest-running-agricultural-experiment). The Hoosfield Spring Barley experiment was established ten years later, in 1853 (Lawes and Gilbert, 1857). The Broadbalk and Hoosfield experiments were established to examine the effects of inorganic fertiliser and organic manures on the grain yields of continuous winter wheat and spring barley, respectively. Grain yields on the experiments have been recorded since the experiments began and weather records have been collected at Rothamsted since the 1850s; records of daily rainfall began in 1853 and temperature records started in 1873. These data, together with detailed information about fertiliser treatments and crop management practices, have been used here to investigate the effects of inter-annual weather variations on crop yield N response for both winter wheat and spring barley.

Previous studies into the effects of weather showed that variations in maximum temperatures in May and June were negatively correlated with wheat grain yields on Broadbalk from 1864 to 1967 (Chmielewski and Potts, 1995) and dry weather was generally beneficial to wheat yields from 1852 to 1918 (Fisher, 1925). However, on the Hoosfield experiment, short periods of heavy summer rainfall benefitted spring barley yields (Wishart and Mackenzie, 1930). More widely, inter-annual variations in rainfall, but not temperature, explained significant levels of wheat yield variability across the Great Plains of the United States of America from 1952 to 2016 (Hatfield and Dold, 2018).

The Rothamsted Long-Term Experiments were not specifically designed to investigate the effect of climate change on crop production, and although informative, previous analyses of the effect of weather on the Rothamsted Long-Term Experiments may be less relevant to the issues relating to climate change and food security in the 21^st^ century. This is because the level of warming experienced in the 21^st^ century has already surpassed that experienced in the late-19^th^ to early- or mid-20^th^ century, the periods considered by Fisher (1921, 1925), Wishart and Mackenzie (1930), and Chmielewski and Potts (1995). However, the yield data and meteorological records associated with these experiments provide an invaluable resource for examining the long-term sustainability of cereal crop production and potential future impacts of climate change (Johnston and Poulton, 2018), and, in particular, the influence of weather on the inter-annual variations in the functional relationship between crop yield and applied N. Focussing particularly on the period since 1968 is also more reflective of current agronomic practices for winter wheat and spring barley.

Grain yield is influenced greatly by N fertilizer and the functional response of crop yield to N application rate varies between years, soil types and crops (Roques et al., 2017; Sylvester-Bradley and Kindred, 2009; Vold, 1998). The relationship between crop yield and applied N can be modelled by a Linear-plus-Exponential (LEXP) function (George, 1982) in which the parameters of the LEXP function can be estimated through a regression framework and the effect of many years’ weather on their estimated values can be assessed.

Here, we define the response curves for the effect of applied N on annual grain yields in winter wheat and spring barley using the LEXP function and the Rothamsted Long-Term datasets. We then investigate the influence of weather variation on these relationships. We test the hypotheses that N yield responses are affected by cultivar, temperature and rainfall systematically – and so can be quantified. Our objective is to understand how variation in weather around different periods within the year have, or have not, altered the response of yield to N for different cultivars of winter wheat and spring barley over the past half a century (1968--2016), in order to identify crop management strategies to help the adaption to future climate change.

## 2 Methods

### 2.1 Rothamsted experimental crop data

The Broadbalk and Hoosfield experiments have been modified throughout their history in order to overcome specific agronomic problems (e.g. weed competition and soil acidification) and to ensure they remain relevant to modern agricultural practices, without losing their long-term integrity (Macdonald et al., 2018).

#### 2.1.1 Broadbalk wheat

**Fig. 1:**
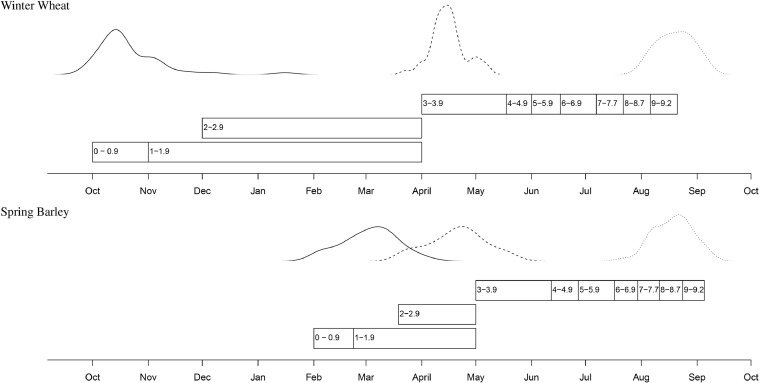
Distribution of sowing (solid line), nitrogen application (kg N ha^−1^; dashed line) and harvest dates (dotted line) for Broadbalk wheat and Hoosfield spring barley. Data includes harvest seasons 1968 to 2016 (2013 and 2015 were excluded from Broadbalk due to late sowing). Also included are the decimal phenological growth stage scores for both cereals (Tottman et al., 1979; Zadock et al., 1974) summarised in Gooding and Davis (1997).

Since 1968, Broadbalk has been comprised of 20 strips (or plots) given different combinations of mineral fertilizers and/or organic manures. The strips were divided into 10 sections (Section 0–9) in 1968 and rotational cropping was introduced on some sections whilst the others remained in continuous wheat. Modern short-strawed wheat cultivars have been grown on the experiment since 1968 with six different cultivars between 1968 and 2016 (Table 1). The grain yield data (at 85% dry matter) for 1968–2016, as used in this study, was from Section 1 (continuous wheat) of Broadbalk; plots 5, 6, 7, 8, 9, 15 and 16 (Macdonald et al., 2018). Sowing of the Broadbalk experiment occurred mostly between October and early-November (Fig. 1). Harvest years 2013 and 2015 were omitted from the analysis, because bad weather delayed sowing from autumn until early spring and a spring wheat variety was therefore used. Harvest year 2001 was retained despite a late sowing date as a winter wheat variety was still used. The plots received adequate mineral fertilizer applications including PKNaMg (Macdonald et al., 2018), plus N applied at rates of 0, 48, 96, 144 or 192 kg N ha^−1^. Additional N application rates of 240 and 288 kg N ha^−1^ were introduced from 1985. The application of N was in a single pass in spring, around early-April with the crop harvested in August and early September (Fig. 1; Macdonald et al., 2018) . The dates of sowing, nitrogen application and harvest for the Broadbalk experiment have remained relatively constant, by design, since the 1968 harvest season (Supplementary Fig. 1 (a-c)).

**Table 1:** Winter wheat cultivars grown in the Broadbalk Long Term Experiment (harvest years 1968 to 2016)

Year	Cultivar
1968–1978	Cappelle Desprez
1979–1984	Flanders
1985–1990	Brimstone
1991–1995	Apollo
1996–2012	Hereward
2014, 2016	Crusoe^1^

1 Sown late in spring 2013 and 2015 and so those years omitted from analysis

#### 2.1.2 Hoosfield spring barley

Spring barley has been grown continuously on the Hoosfield Barley experiment since 1853. The original design of the experiment is of a factorial nature (Warren and Johnston, 1967) with four strips , originally testing four combinations of nutrients: 0 v P v KMgNa v PKMgNa, crossed with four Series testing no N or three forms of N, applied (usually) at 48 kg N ha^−1^ (Series 0, no N; Series A, ammonium sulphate; Series AA, sodium nitrate; Series C, rape cake, later castor meal). Short-strawed cultivars have been grown on the whole experiment since 1968 (Table 2) when most of the existing plots were divided and a four-level N rate application started, replacing the earlier test of different forms of N. Nine cultivars were sown on Hoosfield between 1968 and 2016 (Table 2). In 2003, further changes were made to the experiment. The four-level N rate application continued but P and Mg has been withheld on some plots (and on parts of Series AA) until levels of plant-available P and Mg decline to more appropriate agronomic levels. From 1968 to 2016 barley was usually sown in February with N applied around early-April and the crop harvested around late-August to early-September (Fig. 1). Sowing dates on Hoosfield have been relatively constant since the 1968 harvest season (Supplementary Fig. 1 (d)). However, both nitrogen application, since the mid-00s, and harvest, since the 1990s, have tended to be slightly later than in earlier years (Supplementary Fig. 1 (e, f)).

**Table 2:** Spring barley cultivars grown in the Hoosfield Long Term Experiment (1968 to 2016)

Year	Cultivar
1968–1969	Maris Badger
1970–1979	Julia
1980–1983	Georgie
1984–1991	Triumph
1992–1995	Alexis
1996–1999	Cooper
2000–2007	Optic
2008–2015	Tipple
2016	Irina

Initially (1968–1973) N rates were fixed within each plot but rotated thereafter in the order of 144-96-48-0 kg N ha^−1^, phased in from 1974 to 1980. Barley grain yields (at 85% dry matter) from Series O of Hoosfield with mineral treatments Nil, KNaMg, P or PKNaMg and N applied at 0, 48, 96, 144 kg ha^−1^ from 1968 to 2016 (Macdonald et al., 2018) were analyzed. Yield data was missing for the KNaMg treatment in 2007.

### 2.2 Rothamsted meteorological station data

Monthly mean temperatures (summarised from (daily maximum temperature + daily minimum temperature)/2) and monthly total rainfall for 1967 to 2016 were derived for each month from the Rothamsted Meteorological Station records. All analyses were conducted on a cropping season from October to September each year (e.g. where 2016 represents year of sowing and harvest for spring barley, and harvest year for winter wheat).

Correlations amongst the monthly weather variables (1967–2016) were calculated to assess for collinearities (Fig. 4, Step 4b). Correlations between grain yield (1968–2016) and monthly weather data (1967–2016) were calculated for all N application rates for wheat, and all combinations of N and mineral treatments for spring barley (Fig. 4, Step 4c).

### 2.3 Nitrogen-yield response curve

Commonly, modelling functions including linear-plateau, quadratic, and exponential relationships have been applied to quantify the response of yield (both wheat and barley) to N, but these tend to fit poorly above optimum N rates (Cerrato and Blackmer, 1990). Inverse polynomial functions have also been shown to provide adequate fits for N rate responses (Nelder, 1966). A linear-plus-exponential (LEXP) function (George, 1982) was preferred here because it uses fewer parameters and allows better biological interpretation of parameter estimates. The function for the response of grain yield (t ha^−1^, at 85% dry matter) (*y*) to nitrogen (kg N ha^−1^) (*N*) was:

(1) $$y=a+br^{N}+cN,$$

where *a* is the asymptotic yield (t ha^−1^), *b* is the response of yield to applied N below the optimum (t ha^−1^), *c* is the rate of yield loss from supra-optimal application of N (t ha^−1^), and *r* relates to the curvature of the response. Sylvester-Bradley and Murray (1982) have reported on the efficacy of varying parameter *c* to model yield loss from supra-optimal N.

Nitrogen-yield response curves

(2) $$y_{i}=a_{i}+b_{i}{r_{i}}^{N}+c_{i}N,$$

were fitted to a combined Broadbalk wheat grain yield dataset in response to five (seven from 1985) N levels, for all years (*i*) between 1968 and 2016, initially with separate estimates for parameters *a, b, c* and *r* obtained for each year (i.e. separate response curves for each year) (Fig. 4, Step 2). Further analyses considered models with different parameters, notably *r*, constrained to be common across years (Fig. 4, Step 3), including a single common response curve (i.e. all parameters constrained to be common) fitted across all years (Fig. 4, Step 1).

To allow for the inclusion of the different mineral treatments within the Hoosfield experiment, Eq. (2) was modified for spring barley yield for each year to

(3) $$y_{Ti}=a_{Ti}+b_{Ti}{r_{Ti}}^{N}+c_{Ti}N,$$

where *T* refers to the fertilizer treatments PKNaMg, P, KNaMg, or Nil. This model was fitted to a combined Hoosfield barley grain yield dataset in response to four N levels across all years (i) between 1968 and 2016, again initially with separate parameter estimates for each mineral treatment in each year (i.e. separate response curves for each mineral treatment in each year) (Fig. 4, Step 2). Further analyses again considered models with different parameters, notably *r*, constrained to be common across years (and mineral treatments) (Fig. 4, Step 3), including a single common response curve fitted across all years for each mineral treatment (with *r* constrained to be common across mineral treatments) (Fig. 4, Step 1).

### 2.4 Weather parameterized nitrogen-yield response curve

The non-linear model with separate estimates of all parameters for each year (and mineral treatment) was fitted by the Gauss-Newton method in Genstat® *for Windows* 19th edition (VSN International, 2017). The non-linear model with a common estimate of *r* for all years (and mineral treatments) but separate estimates of the other parameters was fitted similarly. A partial F-test assessed the improvement in fit through allowing separate estimates of *r* compared with a single common estimate (Fig. 4, Step 3)*.*

For the Broadbalk dataset, after assessing the lack of improvement associated with allowing individual estimates of *r* for each year, a maximal model (Eq. (4)) was fitted which included explanatory variables for cultivar (*V*) (i.e. separate parameter estimates for each variety) and monthly weather (*W*, both total monthly rainfall and mean monthly temperature for each of the 12 months from October (sowing) to September (harvest)) fitted as linear functions (f(W+V)) influencing the *a, b* and *c* parameters (Fig. 4, Step 4a)

(4) $$y_{f\left(W+V\right)}=a_{f\left(W+V\right)}+b_{f\left(W+V\right)}r^{N}+c_{f\left(W+V\right)}N$$

Similarly, a maximal model (Eq. (5)) was fitted for the Hoosfield barley dataset with explanatory variables included for cultivar (*V)* and mineral treatment (*T*) (i.e. separate parameter estimates for each variety and each mineral treatment) and weather (*W*, total monthly rainfall and mean monthly temperature for each of the eight months from February (sowing) to September (harvest)), as linear functions (f(W+V+T)) influencing the *a, b* and *c* parameters (Fig. 4, Step 4a)

(5) $$y_{f\left(W+V+T\right)}=a_{f\left(W+V+T\right)}+b_{f\left(W+V+T\right)}r^{N}+c_{f\left(W+V+T\right)}$$

Fixing the non-linear parameter, *r*, of the LEXP function for all years reduced model (variable) selection for Eqs. (4) and (5) to a stepwise multiple linear regression problem.

Before the effects of weather variables were modelled, some explanatory variables were removed due to high collinearity (correlation (|*ρ|*)  > 0.3) with other explanatory variables (see Supplementary Table 1 (a) and (b)) (Fig. 4, Step 4b). For example, if two weather variables were colinear, the variable with the highest mean absolute correlation across all N rates was kept within the maximal model. Those weather variables with the highest mean absolute correlation across all N application rates (Fig. 4, Step 4c) were added into the model first (see Supplementary Table 1 (a) and (b)). Variable selection methods, based on the Akaike Information Criterion (AIC) (Akaike, 1973), were used to reduce the maximal model (after elimination of colinear explanatory variables) by omitting variables one-by-one until the minimal AIC value was obtained, i.e. removing any of the remaining terms did not further reduce the AIC (Fig. 4, Step 5). At each step the variable that most reduced the AIC was omitted.

After the AIC selection procedure, a further model selection process (Fig. 4, Step 6), using partial (marginal) F-tests (the *extra sum of squares principle*), was used to test whether model parameters within the reduced model explained sufficient amounts of model variability (significance level *α* < 0.05) to provide a parsimonious model: a variable was removed if its omission did not significantly reduce the variability explained by the model (i.e. account for significant variability compared with the residual variability) (Welham et al., 2015).

All analyses were applied to square-root transformed yield response data, due to non-constant variance in yields across N rates, with greater variance particularly at higher application rates (Fig. 4, Step 1). All analyses considered the absolute values of yields and weather variables, since the aim of the study was to associate any changes in yield response to N to variation in weather variables (and variety) across years.

Weather-parameterized response curves were fitted using the lm command in R (R Core Team, 2016). Three-dimensional surface plots were produced using the rgl package (Adler and Murdoch, 2016). The CAR package (Fox and Weisberg, 2016) was used to achieve statistical validation of the weather-parameterized models.

## 3 Results

### 3.1 Broadbalk, wheat

#### 3.1.1 Grain yield, 1968 to 2016

**Fig. 2:**
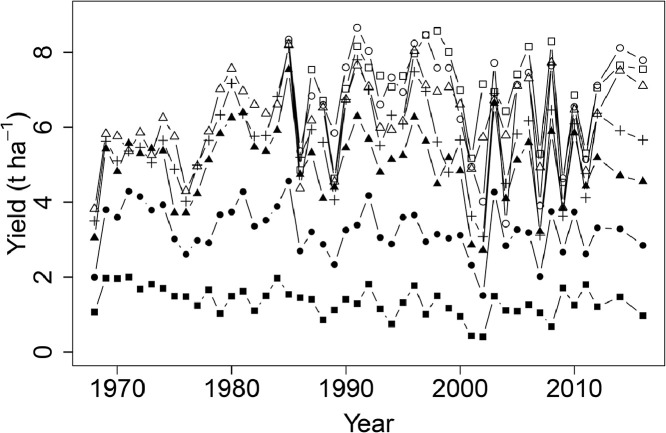
Winter wheat grain yields (t ha^−1^ at 85% dry matter) from Section 1 (continuous wheat with PKNaMg) of the Broadbalk Long Term Experiment from 1968 to 2016 (2013 and 2015 excluded) with nitrogen applied at 0 (▪), 48 (•), 96 (▲), 144 (+), 192 (∆), 240 (○), or 288 (□) kg N ha^−1^.

**Fig. 3:**
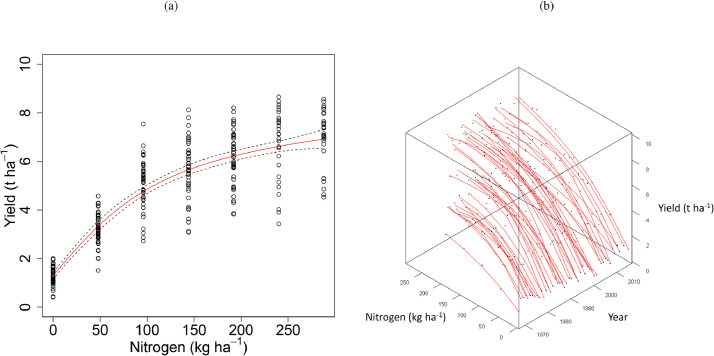
Relationship between winter wheat grain yield (t ha^−1^ at 85% dry matter) and applied nitrogen (kg N ha^−1^) from Section 1 (all with PKNaMg) of the Broadbalk Long Term Experiment provided by the LEXP function for the years 1968 to 2016. (a) LEXP function fitted to data (○) for all years combined (Eq. (1)), with parameter (S.E.) values *a* = 2.402 (0.225), *b* = −1.261 (0.222), *c* = 0.00085 (0.0008), *r* = 0.985(0.003) (fitted curve – red – ±95% Confidence intervals for the curve – dashed). (b) LEXP function fitted to data (•) for each year separately (Eq. (2)), where *r* = 0.988 (S.E. 0.0009) and estimates of *a, b* and *c* varied amongst years. Analyses were conducted on square-root transformed data and the fitted functions back-transformed in the figure.

Mean wheat grain yields were 1.34, 3.26, 5.04, 5.61 and 6.21 t ha^−1^ between 1968 and 2016 for annual applications of 0, 48, 96, 144 and 192 kg N ha^−1^ respectively, and 6.73 and 7.01 t ha^−1^ for annual applications of 240 and 288 kg N ha^−1^ respectively, between 1985 and 2016 (Fig. 2). The variability in yield increased with increasing inputs of N; average variance of annual grain yield between 1985 and 2016 for N treatments up to 144 kg N ha^−1^ was 0.56 t ha^−1^, but for applications above 144 kg N ha^−1^ it was 1.58 t ha^−1^. The common N response curve fitted for grain yield in all years (Fig. 3 (a)) was an asymptotic exponential relationship, with no clear evidence of a decline in yield above an optimum N input.

#### 3.1.2 Nitrogen-yield response curves, individual years

Yield response curves fitted with separate *a, b* and *c* parameters for each individual year (Fig. 3(b)), but with a common value of the non-linear parameter, *r*, estimated at 0.988, explained more variability compared to a single common N response curve (Fig. 3(a)) fitted to all years (F(9.69, 138, 153), P < 0.001). Allowing *r* to vary with year was not necessary as the additional variability was small compared to that for the model with a common estimate of *r* (F(0.392, 46, 107), P = 0.999), in both cases allowing the other parameters (*a, b* and *c*) to vary with year. The period from 1985 onwards generally provided the highest estimated asymptotes for the relationship between yield and N (Fig. 3(b)). The shallowest responses were in 2002 and 2007 with estimated values of *a* of 1.51 and 1.80, and -0.86 and -0.77 for coefficient *b* (Fig. 3(b)). The years 1985 and 1981 provided the highest asymptotic yields in response to N, with estimated values of *a* of 3.60 and 3.42, and -2.37 and -2.15 for coefficient *b*. The greatest loss in yield due to supra-optimal application of N occurred in 1985 (c = -3.77 × 10^−3^), the first year in which the highest N rate was applied.

#### 3.1.3 Correlations between meteorological variables and yield

Generally, the correlation between weather and wheat grain yield was low with only 8 of 84 comparisons reaching significance (α = 0.05) (Supplementary Table 1 (a) and (b)). Correlations between mean January, May and June temperatures, and total October and July rainfall and grain yield were negative for all N application rates, whilst yields for crops given 0, 48 and 96 kg N ha^−1^ were negatively correlated with total October rainfall (Supplementary Table 1). Generally, yields of wheat crops given 240 and 288 kg N ha^−1^ were not correlated with any weather variables at α = 0.05. This indicated that other variables, including cultivar and N application rate, influenced grain yields on Broadbalk to a greater extent than weather from 1968 to 2016.

#### 3.1.4 Weather parameterized nitrogen-yield response curve

**Fig. 4:**
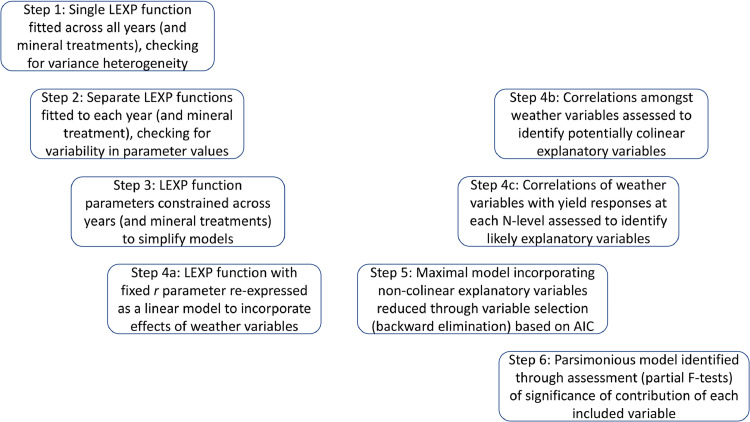
A schematic showing the statistical modelling steps involved in developing parsimonious models to describe the yield response to nitrogen application for both the Broadbalk and Hoosfield Long Term Experiments, including effects of weather variables and variety.

Weather terms (ranked in order of mean absolute correlation between weather and yield across all N application rates, see Fig. 4 for a description of the modelling procedure) within the maximal model were total October rainfall, mean May temperature, total July rainfall, mean November temperature, total February rainfall, mean April temperature, total June rainfall, total December rainfall, total August rainfall, total November rainfall, total May rainfall, mean December temperature and total January rainfall. The weather terms not included above were absent from the maximal model due to high collinearity with those included. The AIC for the maximal model was -977.79 compared to -1017.14 for the reduced model (Table 3). In combination, the weather terms eliminated from the maximal model did not explain a significant amount of variability when compared to the variability explained by the reduced parsimonious weather-parameterized N-yield response model (Table 3) (F(1.42, 40, 272), P = 0.057).

**Table 3:** The final parsimonious model Eq. (4)) for winter wheat grain yield (R^2^ = 89.92%) with model coefficients and standard errors. The non-linear parameter was fixed at *r* = 0.988 (S.E. 0.0009) for all years. Values in the parameter and variable columns refer to weather variables influencing parameters *a, b* and *c* of the LEXP function. This model is presented in a first-level-zero parametrization, with cultivar (cv.) Hereward as the baseline (i.e. the intercept) and the effects of all other cultivars are expressed as the difference from this. Total rainfall and mean temperature are labelled TR and MT, respectively. Terms (1) and (2) refer to the first and second order terms of a quadratic relationship.

Parameter	Variable	Coefficient	S.E.
*a*	Intercept	3.16	0.16
*a*	Cv. Apollo	0.11	0.06
*a*	Cv. Brimstone	0.11	0.06
*a*	Cv. Capelle Desprez	0.15	0.06
*a*	Cv. Crusoe	0.08	0.09
*a*	Cv. Flanders	0.13	0.07
*a*	TR October	-1.65 × 10^−3^	0.26 × 10^−3^
*a*	MT May	−0.04	0.01
*a*	MT November (1)	−0.25	0.24
*a*	MT November (2)	−0.59	0.23
*a*	TR February	−0.58 × 10^−3^	0.36 × 10^−3^
*a*	MT April (1)	−0.80	0.33
*a*	MT April (2)	−1.80	0.31
*a*	TR June	0.61	0.29
*b*	Intercept	−1.55	0.16
*b*	MT April (1)	1.28	0.65
*b*	MT April (2)	2.48	0.57
*c*	Intercept	0.19	0.3
*c*	Cv. Apollo	−0.59 × 10^−5^	0.37 × 10^−3^
*c*	Cv. Brimstone	−0.35 × 10^−3^	0.37 × 10^−3^
*c*	Cv. Capelle Desprez	−2.25 × 10^−3^	0.46 × 10^−3^
*c*	Cv. Crusoe	0.93 × 10^−3^	0.53 × 10^−3^
*c*	Cv. Flanders	−0.43 × 10^−3^	0.55 × 10^−3^

Cultivar was included within the parsimonious model and influenced both the asymptote for yield (*a*) and the rate of yield loss due to supra-optimal application of N (*c*). Cappelle Desprez had the highest estimate of parameter *a* amongst cultivars and the lowest estimate for parameter *c* (Table 3). All cultivars had positive estimates for parameter *c* (values in Table 3 are differences from the Intercept parameter value for Hereward), so there was no evidence of yield loss for supra-optimal application of N.

**Fig. 5:**
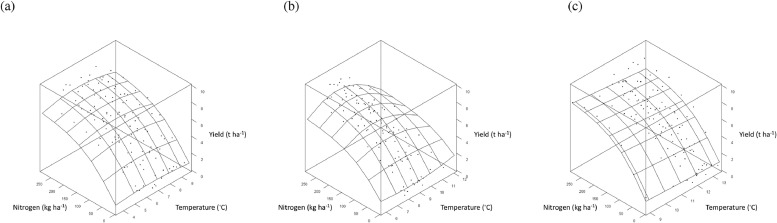
Response surface (Eq. (4), Table 3) of the effect of applied nitrogen (kg N ha^−1^) on winter wheat grain yield (•, t ha^−1^ at 85% dry matter) from Section 1 (all with PKNaMg) of the Broadbalk Long Term Experiment, adjusted for cultivar Hereward, as affected by: (a) Mean November temperature; (b) Mean April temperature; (c) Mean May temperature. Similar 3-dimensional surface plots are provided in Supplementary Figure 1 for the effect of rainfall.

The curvilinear relationship between asymptotic grain yield, *a*, and mean November temperature (Fig. 5 (a)) was quantified with a negative quadratic term (Table 3). Mean November temperature did not affect parameters *b* and *c* and so did not influence the effect of N-rate. The fitted relationship suggests an optimum mean November temperature for wheat grain yield of 6 to 7°C (Fig. 5 (a)). The effect of mean April temperature in the model was also described with a negative quadratic term and influenced both parameters *a* and *b* of the LEXP function (Table 3). Since parameter *b* was affected, the relationship between yield and mean April temperature interacted with the effect of N-rate. The fitted relationship for mean April temperature within the parsimonious model suggests an April temperature of 8 to 8.5°C maximizes grain yield (Fig. 5 (b)). Warmer temperatures in May and greater rainfall in October and February all reduced the value of parameter *a* within the parsimonious model (Table 3), and so resulted in lower asymptotic grain yields throughout the weather ranges studied (e.g. for temperature in May, Fig. 5 (c)). The drier the month of June the lower the asymptote of the N-yield response curve (Table 3), and so the lower the grain yield.

### 3.2 Hoosfield barley

#### 3.2.1 Grain yield, 1968 to 2016

**Fig. 6:**
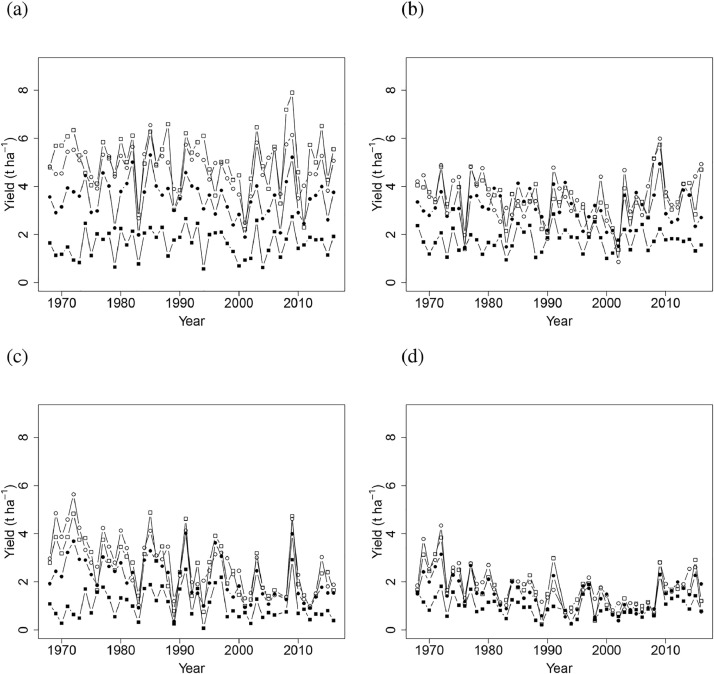
Spring barley grain yield (t ha^−1^ at 85% dry matter) from Series O of the Hoosfield Long Term Experiment from 1968 to 2016 in response to N applied at 0 (▪), 48 (•), 96 (□), 144 (○) kg N ha^−1^ with mineral fertilizer treatments (a) PKNaMg, (b) P, (c) KNaMg, or (d) Nil.

Inter-annual variation from 1968 to 2016 in spring barley grain yield amongst and within the PKNaMg, P, KNaMg, and Nil treatments was considerable (Fig. 6 (a), (b), (c) and (d), respectively). Averaged across all years and N application rates, PKNaMg provided the highest grain yield of 3.73 t ha^−1^, compared to 2.95, 2.13 and 1.48 t ha^−1^ for the P, KNaMg and Nil treatments, respectively. The minimum and maximum mean yields (averaged over N rate treatments) across the different years and fertilizer treatments were 0.55 t ha^−1^ for the Nil mineral treatment in 1994 and 5.50 t ha^−1^ for barley given PKNaMg in 2009.

#### 3.2.2 Common nitrogen response curve

**Fig. 7:**
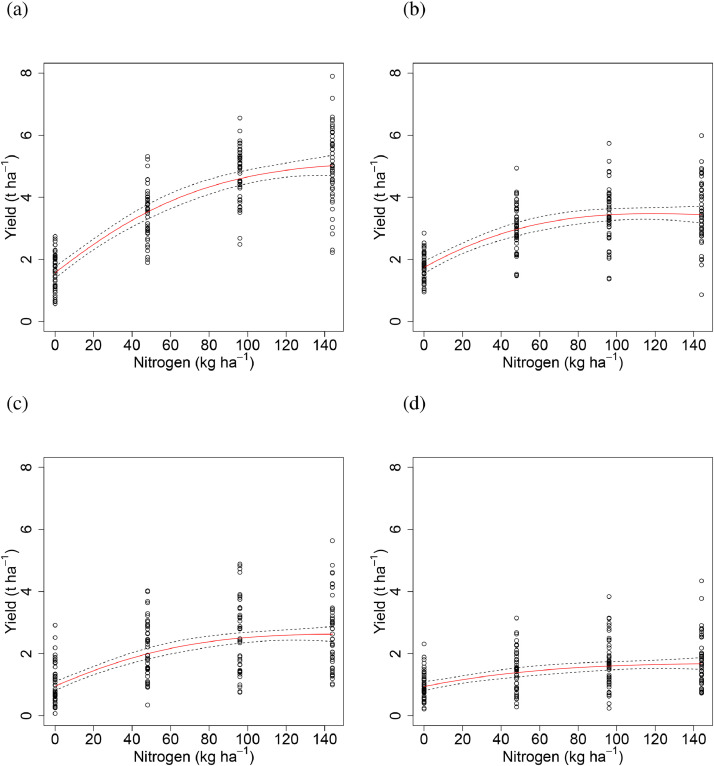
Relationship between spring barley grain yield (○, t ha^−1^ at 85% dry matter) and applied nitrogen (kg N ha^−1^) from Series O of the Hoosfield Long Term Experiment from 1968 to 2016 provided by the LEXP function (fitted curve, Eq. (1)) for mineral fertilizer treatments (a) PKNaMg, (b) P, (c) KNaMg and (d) Nil for all years combined (*r* = 0. 985 (0.0076), fitted curve – red – ±95% Confidence intervals for the curve – dashed). The fitted values of parameters *a, b* and *c* are provided in Supplementary Table 2. Analyses were conducted on the square-root transformed data and the fitted functions back-transformed in this figure.

Allowing the non-linear parameter, *r*, of the LEXP function to vary amongst mineral treatments (Eq. (3)) did not explain any more variation than estimating a common value (*r* = 0.985) for spring barley grain yield in all treatments (F(0.44, 3, 760), P = 0.723), in both cases allowing the other three parameters to vary with mineral treatment. Allowing the *a, b* and *c* coefficients to vary between mineral treatments explained more variability than a common N response curve (F(62.88, 9, 763), p < 0.001). The PKNaMg treatment provided the greatest asymptote (*a* = 2.59) and response to N (*b* = -1.33) (Fig. 7; Supplementary Table 2). The estimated N response curves, averaged over years, were more linear for the KNaMg (Fig. 7 (c)) and Nil (Fig. 7 (d)) treatments than for the PKNaMg (Fig. 7 (a)) and P (Fig. 7 (b)) treatments.

#### 3.2.3 Nitrogen-yield response curves, individual years

Estimating individual parameters *a, b* and *c* for each year and mineral treatment, with a common non-linear parameter, *r*, estimated across both years and mineral treatments, explained more variability than a common curve fitted to all years for each treatment (F(19.27, 579, 193), P < 0.001). The parameter *r* could not be estimated separately for each year because there were only four different N application rates. The common estimate was *r* = 0.989, and so slightly greater than that for all years combined (see Section 3.2.2). Estimating a common *c* for each mineral treatment group explained similar amounts of variability compared to a model estimating an individual *c* parameter for each year and each mineral treatment (F(1.20, 190, 194), P = 0.099). The estimated *c* coefficients for grain yield were -0.00451 (PKNaMg), -0.00483 (P), -0.00398 (KNaMg) and -0.00153 (Nil).

**Fig. 8:**
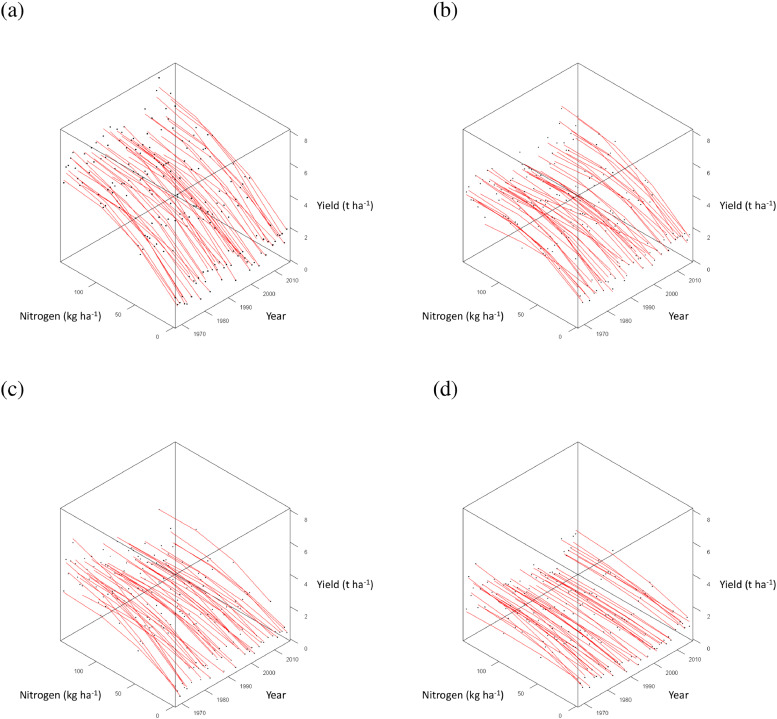
Relationship between spring barley grain yield (•, t ha^−1^ at 85% dry matter) and applied nitrogen (kg N ha^−1^) from Series O of the Hoosfield Long Term Experiment from 1968 to 2016 provided by the LEXP function (fitted curve, Eq. (3)) for each year separately from 1968 to 2016 for mineral fertilizer treatments (a) PKNaMg, (b) P, (c) KNaMg and (d) Nil. Model coefficients are given in Supplementary Table 3. Further details as Fig. 5.

Mineral treatment PKNaMg provided the most well-defined asymptotic relationships of yield with applied N for individual years (Fig. 8 (a)). The N response curves tended to be more linear and flatter (lower yield) amongst the remaining mineral treatments P (Fig. 8 (b)), KNaMg (Fig. 8 (c)), Nil (Fig. 8 (d)). For mineral treatment PKNaMg, 2009 provided the largest estimate of *a* (3.82), the asymptote, compared to the lowest (2.51) in 2001; and 1994 provided the largest estimate of *b* (-3.01), the yield response to N, with the smallest estimate in 2011 (-1.38).

#### 3.2.4 Correlations between meteorological variables and yield

Grain yield from all N and mineral treatments showed a consistent negative correlation with April rainfall (Supplementary Table 3 (a)), whereas for June rainfall the correlation was consistently positive.

There were weak correlations between grain yield and the monthly temperature variables for all N treatments with mineral treatment PKNaMg. The correlations between spring barley yield and May, June and July temperatures were negative for all N and mineral treatments (Supplementary Table 3 (b)). May and July temperatures had negative correlations with grain yield at every N application rate for the KNaMg and Nil mineral treatments. Grain yield from plots given 48, 96 and 144 kg N ha^−1^ plus KNaMg and those without minerals (Nil) had a negative correlation with June temperature.

#### 3.2.5 Weather parameterized nitrogen-yield response curve

The AIC of the maximal model (Eq. (5)) for spring barley grain yield was -2204.5 compared to -2255.3 for the reduced model. The maximal model for spring barley (Eq. 4) did not explain a significant additional amount of variability when compared to the reduced parsimonious weather-parametrized N-response model (F(1.19, 84, 563), P = 0.136)). Terms fitted within the maximal model included mineral treatment and cultivar as factor (qualitative) explanatory variables along with the weather variables mean June temperature, total April rainfall, mean February temperature, total May rainfall, total September rainfall and total July rainfall. Yields from years 1993, 1998, 2000 and 2012 were omitted from the spring barley parsimonious model, due to extreme April rainfall values inverting the usual negative quadratic relationship to be a positive one, producing an unrealistic model driven by outliers.

In the parsimonious model, the terms for mineral treatment and cultivar influenced the asymptote (*a*) and the magnitude of the yield response to N (*b*). Estimates of *a* for mineral treatments P and Nil were 0.43 and 0.29 greater, respectively, than those for PKNaMg (Table 4). Similarly, estimates of *b* were 0.55 and 0.84 greater for treatments P and Nil than for PKNaMg. The greater estimates of both *a* and *b* for mineral treatments P and Nil suggest that crops grown under these two mineral treatments were less efficient than those grown under the PKNaMg treatment at utilizing N at lower rates.

**Table 4:** The final parsimonious model Eq. (5)) for spring barley grain yield (R^2^ = 83.17%) with model coefficients and standard errors. The non-linear parameter was fixed at *r* = 0.989 (S.E. 0.0025) for all mineral treatments and years. Values in the in the parameter and variable columns refer to weather variables influencing parameters *a, b* and *c* of the LEXP function. This model is presented in a first-level-zero parametrization, with cultivar (cv.) Tipple and Treatment (Treat.) PKNaMg as the baseline (i.e. the intercept) with the effects of all other cultivars and mineral fertilizer treatments (KNaMg, P, or Nil) expressed as the difference from this. Total rainfall and mean temperature are labelled TR and MT, respectively. Terms (1) and (2) refer to the first and second order terms of a quadratic relationship.

Parameter	Variable	Coefficient	S.E.
*a*	Intercept	3.27	0.25
*a*	Treat. P	0.43	0.31
*a*	Treat. KNaMg	−0.03	0.31
*a*	Treat. Nil	0.29	0.31
*a*	Cv. Alexis	0.22	0.1
*a*	Cv. Cooper	−0.19	0.09
*a*	Cv. Georgie	0.16	0.09
*a*	Cv. Irina	0.18	0.15
*a*	Cv. Julia	0.17	0.07
*a*	Cv. Maris Badger	0.09	0.12
*a*	Cv. Optic	−0.18	0.07
*a*	Cv. Triumph	0.07	0.07
*a*	MT June	0.01	0.01
*a*	TR April (1)	−2.57	0.43
*a*	TR April (2)	−3.08	0.42
*a*	MT February	−0.04	0.01
*a*	TR September	−2.01 × 10^−3^	0.44 × 10^-3^
*a*	Treat. P: Cv. Alexis	−0.14	0.1
*a*	Treat. KNaMg: Cv. Alexis	−0.08	0.1
*a*	Treat. Nil: Cv. Alexis	−0.5	0.11
*a*	Treat. P: Cv. Cooper	−0.16	0.1
*a*	Treat. KNaMg: Cv. Cooper	0.36	0.1
*a*	Treat. Nil: Cv. Cooper	0.07	0.1
*a*	Treat. P: Cv. Georgie	−0.28	0.09
*a*	Treat. KNaMg: Cv. Georgie	0.1	0.09
*a*	Treat. Nil: Cv. Georgie	−0.22	0.09
*a*	Treat. P: Cv. Irina	−0.06	0.15
*a*	Treat. KNaMg: Cv. Irina	−0.13	0.15
*a*	Treat. Nil: Cv. Irina	−0.15	0.15
*a*	Treat. P: Cv. Julia	−0.16	0.07
*a*	Treat. KNaMg: Cv. Julia	0.31	0.07
*a*	Treat. Nil: Cv. Julia	0.09	0.07
*a*	Treat. P: Cv. Maris Badger	3.11 × 10^−3^	0.12
*a*	Treat. KNaMg: Cv. Maris Badger	0.33	0.12
*a*	Treat. Nil: Cv. Maris Badger	0.22	0.12
*a*	Treat. P: Cv. Optic	−0.09	0.08
*a*	Treat. KNaMg: Cv. Optic	0.1	0.08
*a*	Treat. Nil: Cv. Optic	−0.12	0.08
*a*	Treat. P: Cv. Triumph	−0.32	0.08
*a*	Treat. KNaMg: Cv. Triumph	0.14	0.08
*a*	Treat. Nil: Cv. Triumph	−0.31	0.08
*a*	Treat. P: MT June	−0.09	0.02
*a*	Treat. K Na Mg: MT June	−0.06	0.02
*a*	Treat. Nil: MT June	−0.05	0.02
*a*	Treat. P: TR April (1)	−0.06	0.6
*a*	Treat. KNaMg: TR April (1)	−1.22	0.62
*a*	Treat. Nil: TR April (1)	1.72	0.6
*a*	Treat. P: TR April (2)	2.72	0.6
*a*	Treat. KNaMg: TR April (2)	1.12	0.63
*a*	Treat. Nil: TR April (2)	1.17	0.6
*b*	Intercept	−2.07	0.14
*b*	Treat. P	0.55	0.07
*b*	Treat. KNaMg	0.42	0.07
*b*	Treat. Nil	0.84	0.07
*b*	Cv. Alexis	−0.26	0.13
*b*	Cv. Cooper	0.18	0.12
*b*	Cv. Georgie	−0.06	0.11
*b*	Cv. Irina	−0.15	0.18
*b*	Cv. Julia	−0.3	0.08
*b*	Cv. Maris Badger	−0.44	0.14
*b*	Cv. Optic	0.16	0.09
*b*	Cv. Triumph	0.08	0.09
*b*	TR September	2.78 × 10^−3^	0.7 × 10^−3^
*c*	Intercept	−3.91 × 10^−3^	0.64 × 10^−3^

Among the nine cultivars, Alexis provided the largest estimate of *a*: 0.22 greater than Tipple and 0.41 greater than Cooper, the third-lowest and lowest asymptotes respectively (Table 4). The estimate of *b* for Alexis was also lower than for Tipple. These values suggest that Alexis was more efficient at utilizing N as application rate increased and had a greater asymptotic yield than Tipple. Maris Badger provided the largest negative estimate of *b* (values in Table 4 are differences from the Intercept value for Tipple), suggesting it was the most efficient cultivar at utilizing N as application rate increased (a more negative value of *b* indicates a bigger increase in yield between 0 kg N ha^−1^ and the asymptotic yield estimate). There was evidence to suggest an interaction between mineral treatment and cultivar on the value of parameter *a* (hence the variant values in Table 4) suggesting cultivars responded differently to the mineral treatments, as well as to applied N.

**Fig.9:**
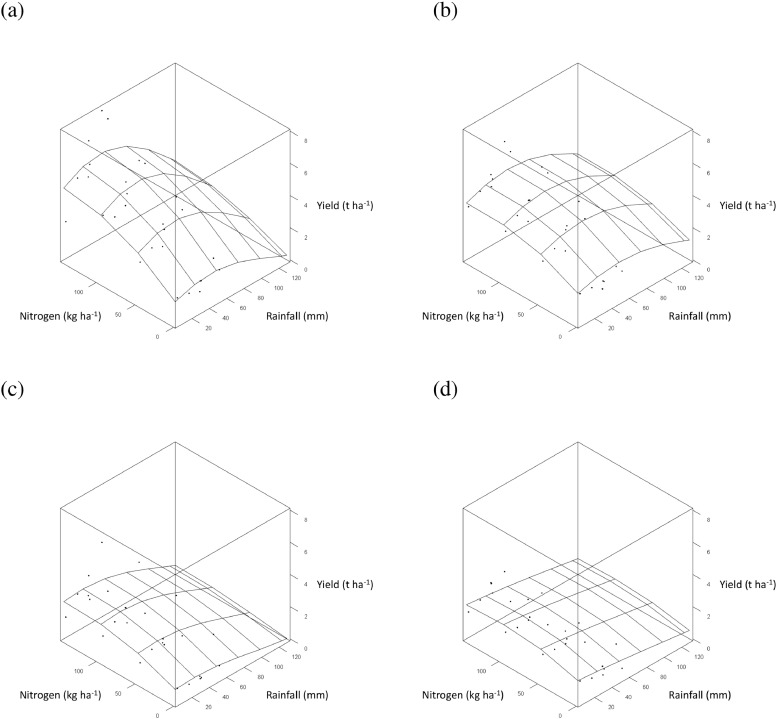
Response surface (Eq. (5), Table 4) for the effect of applied nitrogen (kg N ha^−1^) on spring barley grain yield (•, t ha^−1^ at 85% dry matter), adjusted for cultivar Tipple, as affected by mean April rainfall for mineral fertilizer treatments (a) PKNaMg, (b) P, (c) KNaMg and (d) Nil. Further details as Fig. 5.

In the parsimonious model, several weather variables influenced estimates of *a*, only one affected those of *b*, and none affected those of *c* (Table 4). Total rainfall in April had the largest impact on grain yield amongst weather variables and influenced estimates of *a*. The effect on *a* was quantified by a negative quadratic relationship (Table 4). Total rainfall in April also interacted with mineral treatment. Treatment PKNaMg provided the largest negative estimate of the second order polynomial term affecting *a* (Table 4), whereby excess rainfall in April reduced yield more severely for the higher mineral fertilizer inputs (decline in yield in PKNaMg > KNaMg >P >Nil; Fig. 9 (a)–(d)).

**Fig. 10:**
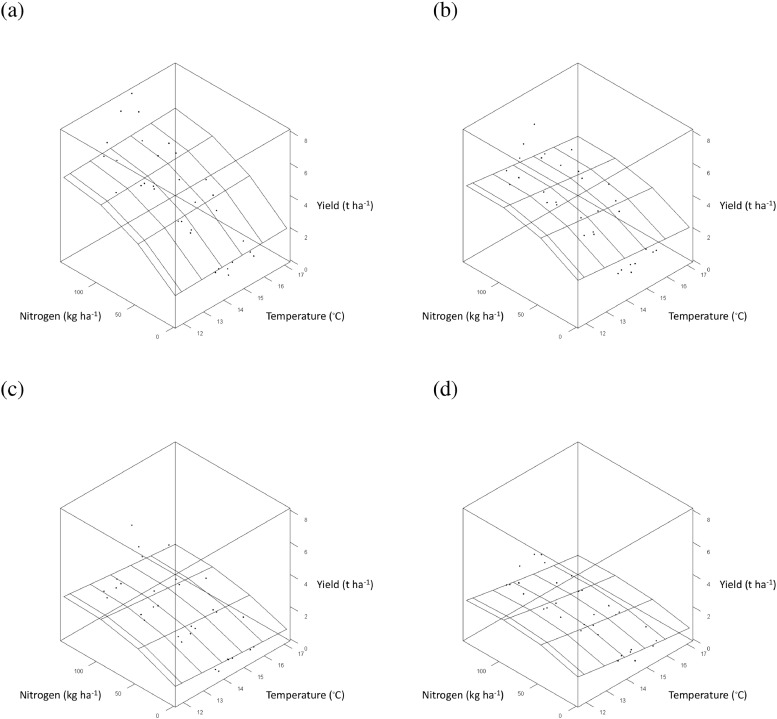
Response surface (Eq. (5), Table 4) for the effect of applied nitrogen (kg N ha^−1^) on spring barley grain yield (•, t ha^−1^ at 85% dry matter), adjusted for cultivar Tipple, as affected by mean June temperature for mineral fertilizer treatments (a) PKNaMg, (b) P, (c) KNaMg and (d) Nil. Further details as Fig. 5.

High temperatures in June reduced the estimate of *a* for mineral treatments P, KNaMg and Nil within the parsimonious model (Table 4; Fig. 10 (b)–(d)), but this was not evident in PKNaMg (Table 4, Fig. 10 (a)). Mean February temperature reduced *a* (Table 4), but no interaction with mineral treatment was detected (Supplementary Fig. 3). September rainfall was the only weather variable to influence estimates of *b*, with an increased response of yield to N with lower levels of rainfall (Table 4) and a more linear N response with more rainfall in this month (Supplementary Fig. 4). Higher total rainfall in September reduced estimates of *a*, however (Table 4).

## 4 Discussion

Weather-parameterized LEXP functions (Eqs. (4) and (5)) were applied successfully to selected data from two long-term experiments to quantify the effect of weather and cultivar on the N-yield response for both winter wheat and spring barley. This approach provides a greater understanding of the influence of weather on the grain yields of these crops in the UK. Inter-annual variability in the N-yield response curves was explained by variations in weather at different times of the year, and by cultivar, in each crop, with additional effects of mineral treatments in barley. Our research builds upon previous studies of the influence of climate and weather on grain yield from the Rothamsted Long-Term Experiments (Chmielewski and Potts, 1995; Fisher, 1925, 1921; Lawes and Gilbert, 1880, 1871; Wishart and Mackenzie, 1930); upon studies where N-yield response curves were shown to vary with year, soil and weather (Roques et al., 2017; Sylvester-Bradley and Kindred, 2009; Vold, 1998); and upon studies where wheat yield in dry (cf. wet) years have been shown to have limited or no positive response to applied N (Ellis et al., 1999; Gooding et al., 1993).

The absence of many weather variables from the maximal and, especially, the parsimonious models should not be overstated. The lack of significant correlations (α=0.05) between many weather variables and grain yield does not imply they have no effect. The large variability in the bivariate relationship between weather and yield from the long-term experiments may result in non-significant relationships being reported at the traditional 0.05 significance level, especially at higher N application rates where yield variability is large. At a broader level, all weather contributes to yield – for example, intercepted radiation each day (Monteith, 1977). The use of levels of statistical significance in the context of the binary and potentially misleading conclusions proposed in some previous studies has been discussed recently by Amrheim et al. (2019), with whom we concur in this application.

The parsimonious model for N-yield response curves for winter wheat from 1968 to 2016 (Eq. (4)) required only six temporal weather variables (at the level of significance applied here): negative effects of total rainfall in October and February, but a positive effect for total rainfall in June; and a curvilinear response to mean temperature in November and April, whereby optimal temperatures were detected, and a negative effect of mean temperature in May. The maximal winter wheat model included a further six effects: some were the other weather variable for the above months, viz. total rainfall in November and May, and others were weather variables in neighbouring months, viz. both rainfall and temperature in December, and rainfall in January and August. In spring barley, similarly to winter wheat, the parsimonious model for N-yield response curve from 1968 to 2016 (Eq. (5)) required only five temporal weather variables (rainfall in April and September; mean temperature in February, June and September). We suggest that the collinearity of temporal weather variables explains both the above variable selections and the absence of any effects for the remaining temporal weather variables investigated (such as rainfall in April). Also, the building of the maximal model and the ranking of model terms before the backwards AIC variable selection process was based upon the mean correlation of all N levels with each weather variable, respectively. The omission of variables due to collinearity may therefore be dependent on the ranking of model terms in the initial maximal model. This is a limitation of the statistical modelling approach used within a regression framework here. The issue of collinearity has been raised in other studies assessing the impact of climate change on food production (Katz, 1977).

The temporal weather variables identified in the winter wheat and spring barley parsimonious models relate well to crop phenology and agronomy (Fig. 1): they coincide with the timings of sowing and subsequent seedling establishment, N application, anthesis and early grain filling in both crops, and also of late grain filling in barley (Gooding and Davis, 1997; Tottman et al., 1979; Zadock et al., 1974). Wetter and cooler conditions around sowing have been shown in previous studies to reduce grain yield for both of these cereals on the Rothamsted Long-Term Experiments (Fisher, 1925; Wishart and Mackenzie, 1930). In wheat, cold and wet conditions delay and reduce seedling emergence (Khah et al., 1986), resulting in lower plant population density and reduced grain yield (Ellis et al., 1999; Gooding et al., 2002; Khah et al., 1989). High rainfall in spring, soon after N application, can contribute to losses of fertilizer N (Powlson et al., 1992, 1986). Spring cereals in particular may be more susceptible to losses of N by leaching (Allingham et al., 2002) and denitrification (gaseous N loss) soon after N application (Addiscott and Powlson, 1992) because of their limited root development (Glendining et al., 1997). The model complexity illustrates how variations and changes in weather variables, such as temperature and rainfall, during sensitive stages of crop development within a year influence the yield response to nitrogen application, as summarized by impacts of weather variables on the *a, b* and *c* parameters of the LEXP function separately.

Analysis of Broadbalk winter wheat grain yield before 1968 found a negative correlation with May and June temperatures (Chmielewski and Potts, 1995). This is compatible with the current analysis from 1968 onwards where there was a negative effect for May temperature and a positive one for June rainfall, given a negative association between temperature and rainfall in June. High temperatures around anthesis are known to reduce grain yield in wheat due to poorer seed set (Ferris et al., 1998; Wheeler et al., 1996), and warmer temperatures and drought after anthesis reduce grain filling (Gooding et al., 2003). Therefore, the effect of climate change on crop growth is not constant throughout the year and is dependent on environmental conditions at specific within-year stages of crop development (Fig. 1). To maximise future yields, the effects of month-by-month changes in climate on yield response to N in cereals have to be considered, and mitigated through manipulation of growing conditions and/or breeding of new varieties with appropriate traits.

As well as the collinearity of temporal weather variables, there is further confounding of variables that handicaps the accurate identification of sources of variability within these long-term yield datasets. For example, the higher applications of N in the Broadbalk Experiment have resulted in a slightly higher soil organic carbon content than for lower rates (Johnston et al., 2009; Watts et al., 2006) which may influence the parameterization of N-yield response curves. Also, atmospheric CO_2_ concentration has increased progressively between 1968 and 2016 (NOAA, 2018). This is especially pertinent to the estimation of the effects of the different cultivars, which were grown in different limited periods (Tables 1 and 2) and so under different atmospheric CO_2_ concentrations.

While cultivar explained sufficient amounts of model variability within the parsimonious models for winter wheat and spring barley, estimates of coefficients for cultivar may be confounded with differences in weather, pests and disease (Glynne, 1969) and their control, as well as atmospheric CO_2_ concentration, particularly given the warming experienced over this period from 1968 to 2016 where extreme weather resulted in outliers. This confounding was a greater issue in the Hoosfield spring barley experiment, with nine different cultivars (Table 2), than in the Broadbalk winter wheat experiment, with only six (Table 1). For example, Alexis and Cooper were only sown in Hoosfield for four years each. An extreme outlier led to an inappropriate estimation of regression model coefficients and so an outlier year was omitted for each. Similarly, two years of data (2013 and 2015) were excluded on Broadbalk because of late sowings, with only two years’ data remaining from which coefficients were estimated for Crusoe. Observations for Irina (Hoosfield), moreover, were limited to a single year. Nonetheless, due to the assumption of a common impact of weather variables on LEXP parameters being estimated across cultivars, preliminary estimates of model coefficients could be estimated for Irina.

Hence careful consideration of the different inter-annual variation in weather and atmospheric CO_2_ concentration that cultivars have been exposed to is required when considering the potential interpretation of different estimates of model parameters amongst cultivars obtained from long-term agricultural experiments. Nonetheless, it is known from other studies that cultivars may differ in optimum N application rates, e.g. in triticale (x*Triticosecale rimpaui* Wittm.) (Roques et al., 2017), and in asymptotic yield and sensitivity of yield to N, e.g. in winter wheat (Gooding and Davies, 1997; Ellis et al., 1999), as shown here for both winter wheat (Table 3) and spring barley (Table 4).

**Fig. 11:**
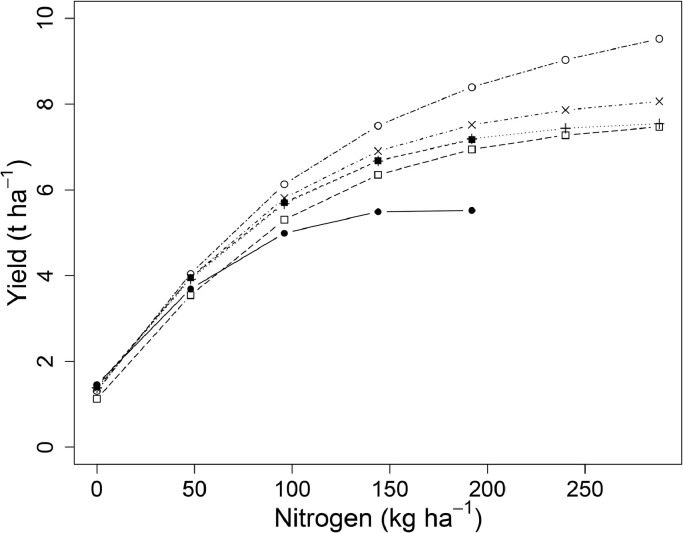
Estimated nitrogen response curves for each cultivar on Broadbalk from 1968 to 2016 (Table 1) from the parsimonious model (Table 3) for grain yield. Cappelle Desprez (•), Flanders (▪), Brimstone (+), Apollo ( × ), Hereward (□) and Crusoe (○). The means of all other weather variables were used in this prediction.

**Fig. 12:**
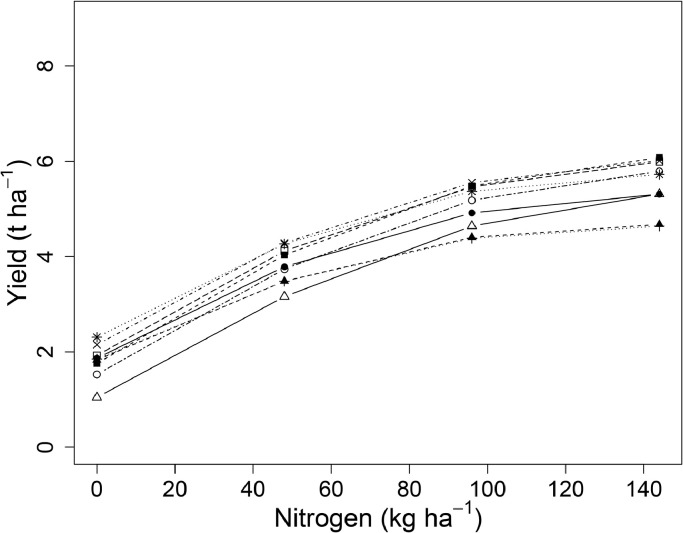
Estimated nitrogen response curves for each cultivar on Hoosfield from 1968 to 2016 (Table 2) from the parsimonious model (Table 4) for grain yield. Maris Badger (Δ), Julia (○), Georgie ( × ), Triumph (*), Alexis (▪), Cooper (+), Optic (▲), Tipple (•) and Irina (□). The means of all other weather variables were used in this prediction.

Hence, whilst a degree of caution is required when applying the parameter estimates obtained, the analytical approach outlined here (Eqs. (4) and (5)) can provide a greater understanding of the inter-relationships between weather, cultivar, and the response of yield to applications of N – and, moreover, quantify them. Our analyses provide a basis on which varietal improvements in response to N fertilizer and variation in weather can be examined over the past half century. The former is an important agronomic input for yield, of course. In addition, N use efficiency varies amongst genotypes and improving the use of N fertilizer is important because it is causes more than half of the greenhouse gas emissions associated with wheat production (Gaju et al., 2011). The modern wheat cultivar Crusoe (Table 1) was identified as the highest yielding cultivar grown on Broadbalk through the parsimonious model (Table 3), and responded best to higher rates of N. In contrast, Cappelle Desprez, the oldest of the short-strawed cultivars, was identified as the lowest yielding and least responsive to N (Fig. 11). Similarly, in spring barley, the newest cultivar (Irina, Table 2) was identified as one of the highest yielding varieties. However, with the spring barley cultivars sown on Hoosfield there does not seem to be a clear improvement in yield with more modern cultivars (Fig. 12). This conclusion may be limited by the frequent changes in cultivar over time, and the extreme weather conditions within one year for a cultivar which was sown only for a few years, thereby confounding cultivar estimates. The extent of genetic improvements in cultivars since the 1960s may also be overestimated or confounded (Figs. 9 and 10) due to the increase in atmospheric carbon dioxide concentration over this period (IPCC, 2014) (Tables 1 and 2).

Before this work we would have advocated selection of wheat varieties with better disease resistance and better resistance to lodging in order to support earlier sowing in autumn, which improves natural uptake of N from soil, and so early canopy development, and reduces nitrogen leaching from soil early in the growing season (Gooding and Davies, 1997). Based on the current results and anticipating future climate change, we would also now suggest that plant breeders select future UK wheat cultivars for high grain yield, with no diminution of protein concentration in the grain, at high levels of nitrogen application (i.e. optimal values of 240–288 kg N ha^−1^) combined with (a) warmer temperatures in April (Table 3, Fig. 4) and/or (b) drier conditions in June (Table 3, Supplementary Fig. 2). These environmental modifications can be achieved in field plots using temporary polythene covers and rain-out shelters, respectively. Selection is suggested at high nitrogen application rates to maximise grain yield in these circumstances, but also because if environmental regulations limit nitrogen application rates in future then the current winter wheat results (Fig. 11) show that varieties which provide greater yield at high nitrogen application rates (240–288 kg N ha^−1^) also provide greater yield at intermediate rates (e.g. 96–144 kg N ha^−1^). Similar breeding strategies can be determined for spring barley, though the greater complexity of the parsimonious model (Table 4) means that a more complex combination of temperature and rainfall controls is needed alongside consideration of mineral treatments.

The analyses within this study also assess the effect of climate change on the Rothamsted Long-Term Experiments. Previous studies indicated that rainfall explained more of the yield variations in wheat compared with temperature (Hatfield and Dold, 2018). Similarly, previous analyses of yield data from the Rothamsted Long-Term Experiments by Lawes and Gilbert (1880, 1871), Fisher (1925, 1921), and Wishart and Mackenzie (1930), focused more on the effect of rainfall than of temperature. However, the increasing trend in annual temperature at Rothamsted and elsewhere observed since the latter part of the 20^th^ century (IPCC, 2014) has highlighted the importance of the effect of temperature and enhanced atmospheric CO_2_ on crops grown in the Long-Term Experiments. By examining N-yield responses for crops in two of these experiments we have shown that the separate effects of management and climate on crop production can be identified.

## 5 Conclusions

The major conclusions of this study are:

(1) The LEXP function successfully quantified N-yield responses for winter wheat and spring barley using records of grain yield from two long-term experiments at Rothamsted over the period 1968 to 2016.
(2) This approach quantified the effects of crop management and of weather on yield, and so provides a greater understanding of the influence of weather, cultivar, and applied N on crop yield.
(3) Significant variability in N yield response curves was explained by inter-annual variation in weather at different times of the year and by cultivar in each crop.
(4) Temporal weather variables show collinearity and so comparatively few were required in parsimonious models.
(5) Weather at key stages of crop development had the greatest effect on N-response curves. Particularly weather-sensitive times coincided with sowing, N application, and anthesis and grain filling.
(6) Collinearity of weather variables and over reliance on the statistical significance level of α=0.05 may result in an underestimation of the effect of weather throughout the cropping season on the response of crop yields to N.
(7) Plant breeding over the past half a century has improved yield and the response of yield to N fertilizer in winter wheat but there is a lack of evidence for this amongst the spring barley cultivars sown on the Rothamsted Long-Term Experiments.

## Declaration of Competing Interest

We wish to confirm that there are no known conflicts of interest associated with this publication and there has been no significant financial support for this work that could have influenced its outcome.

## Appendix Supplementary materials

Image, application 1

Supplementary Fig. 1. The sowing (a), nitrogen application (b) and harvest dates (c) for the Broadbalk wheat experiment. The sowing (d), nitrogen application (e) and harvest dates for the Hoosfield spring barley experiment. Data includes harvest seasons 1968 to 2016 (2013 and 2015 were excluded from Broadbalk due to late sowing). The solid line represents a five-year rolling mean, with dates expressed relative to the 1^st^ of October for each year. Image, application 2

Supplementary Figure 2. Response surface (Equation (4), Table 3) of the effect of applied nitrogen (kg N ha^−1^) on winter wheat grain yield (•, t ha^−1^ at 85% dry matter) from Section 1 (all with PKNaMg) of the Broadbalk Long Term Experiment, adjusted for cultivar Hereward, as affected by: (a) Total October rainfall; (b) Total February rainfall; (c) Total June rainfall. Image, application 3

Supplementary Figure 3. Response surface (Equation (5), Table 4) for the effect of applied nitrogen (kg N ha^−1^) on spring barley grain yield (•, t ha^−1^ at 85% dry matter), adjusted for cultivar Tipple, as affected by mean February temperature for mineral fertilizer treatments (a) PKNaMg, (b) P, (c) KNaMg, or (d) Nil. Image, application 4

Supplementary Figure 4. Response surface (Equation (5), Table 4) for the effect of applied nitrogen (kg N ha^−1^) on spring barley grain yield (•, t ha^−1^ at 85% dry matter), adjusted for cultivar Tipple, as affected by September rainfall for mineral fertilizer treatments (a) PKNaMg, (b) P, (c) KNaMg, or (d) Nil. Image, application 5

## Footnotes

Supplementary material associated with this article can be found, in the online version, at doi:10.1016/j.agrformet.2019.107898.
