# Supplementary material for: Investigating the effects of inter-annual weather variation (1968–2016) on the functional response of cereal grain yield to applied nitrogen, using data from the Rothamsted Long-Term Experiments
Source: Agric For Meteorol. 2020 Apr 15;284:107898. doi: 10.1016/j.agrformet.2019.107898 (PMC7079297; doi:10.1016/j.agrformet.2019.107898)
Supplement: Supplementary file 1 [file mmc1.docx]

Supplementary Table 1: Pearson’s correlation coefficient between winter wheat grain yield at different Nitrogen levels, and summarized monthly total rainfall (a) and mean temperature (b). (* P < 0.050)

| **(a)** |  |  |  |  |  |  |  |  |  |  |  |  |
| --- | --- | --- | --- | --- | --- | --- | --- | --- | --- | --- | --- | --- |
| **Total Rainfall (mm)** | **October** | **November** | **December** | **January** | **February** | **March** | **April** | **May** | **June** | **July** | **August** | **September** |
| **0 Kg N ha^-1^ PKNaMg** | -0.4846* | -0.0207 | -0.0835 | -0.0552 | -0.1455 | -0.1392 | -0.2319 | -0.1360 | 0.0732 | -0.2726 | -0.2042 | -0.0356 |
| **48 Kg N ha^-1^ PKNaMg** | -0.3641* | 0.0640 | 0.1013 | -0.0438 | -0.2072 | 0.1559 | -0.1179 | 0.0371 | 0.0765 | -0.2761 | -0.0957 | -0.0551 |
| **96 Kg N ha^-1^ PKNaMg** | -0.3437* | -0.0925 | 0.1793 | -0.1514 | -0.2245 | 0.0338 | -0.1271 | -0.0339 | 0.1583 | -0.1932 | -0.1107 | -0.2083 |
| **144 Kg N ha^-1^ PKNaMg** | -0.2254 | -0.1267 | 0.2337 | -0.0637 | -0.1217 | 0.0211 | -0.0560 | -0.0276 | 0.1537 | -0.1329 | -0.1129 | -0.1035 |
| **192 Kg N ha^-1^ PKNaMg** | -0.0659 | -0.0943 | 0.1430 | 0.0155 | -0.0561 | 0.1049 | -0.0426 | 0.0792 | 0.0933 | -0.0527 | 0.0029 | -0.1016 |
| **240 Kg N ha^-1^ PKNaMg** | -0.0913 | -0.1842 | 0.0509 | -0.0456 | -0.1299 | -0.2442 | -0.0730 | -0.1948 | 0.2158 | -0.2215 | -0.1342 | 0.1910 |
| **288 Kg N ha^-1^ PKNaMg** | -0.0678 | -0.0188 | -0.0505 | -0.0711 | -0.2007 | -0.1889 | -0.0386 | 0.0448 | 0.2283 | -0.0702 | -0.0511 | 0.2901 |
|  |  |  |  |  |  |  |  |  |  |  |  |  |
| **(b)** |  |  |  |  |  |  |  |  |  |  |  |  |
| **Mean Temperature (°C)** | **October** | **November** | **December** | **January** | **February** | **March** | **April** | **May** | **June** | **July** | **August** | **September** |
| **0 Kg N ha^-1^ PKNaMg** | -0.0205 | -0.1292 | -0.1865 | -0.1352 | -0.2740 | -0.3618* | -0.1245 | -0.1987 | -0.1234 | -0.1452 | -0.1454 | -0.0874 |
| **48 Kg N ha^-1^ PKNaMg** | -0.1193 | 0.1922 | -0.0553 | -0.2546 | -0.2394 | -0.2570 | -0.2041 | -0.1813 | -0.2444 | -0.0308 | -0.0754 | -0.1531 |
| **96 Kg N ha^-1^ PKNaMg** | 0.0397 | 0.1456 | -0.0963 | -0.3547* | -0.3057* | -0.1327 | -0.1705 | -0.2419 | -0.2350 | 0.0339 | -0.0427 | -0.0085 |
| **144 Kg N ha^-1^ PKNaMg** | 0.0509 | 0.2036 | 0.0076 | -0.2875 | -0.2288 | -0.0651 | -0.1999 | -0.2945* | -0.2281 | 0.0394 | 0.0579 | -0.0740 |
| **192 Kg N ha^-1^ PKNaMg** | 0.1646 | 0.3368* | 0.0773 | -0.0744 | 0.0495 | 0.0326 | 0.0405 | -0.0691 | -0.0931 | 0.0900 | 0.0929 | 0.2317 |
| **240 Kg N ha^-1^ PKNaMg** | 0.0425 | -0.0417 | -0.0431 | -0.1671 | -0.1585 | 0.0565 | -0.2355 | -0.2209 | -0.2366 | 0.2180 | 0.0343 | 0.0088 |
| **288 Kg N ha^-1^ PKNaMg** | 0.1256 | 0.1780 | -0.1747 | -0.0562 | 0.0643 | -0.0050 | -0.0202 | -0.2184 | -0.1679 | 0.0786 | 0.0750 | 0.1263 |

Supplementary Table 2: Estimated model coefficients (and standard errors) of the LEXP function (a, b, c; Equation (3)) for grain yield of spring barley, fitted separately to each mineral fertilizer treatment group. The non-linear parameter was fixed at r = 0.985 (S.E. 0.0076) for all four treatments. The fitted lines are presented in Figure 5.

|  | **PKNaMg** | **P** | **KNaMg** | **Nil** |
| --- | --- | --- | --- | --- |
| a | 2.59 (0.18) | 2.33 (0.25) | 1.98 (0.25) | 1.42 (0.25) |
| b | -1.33 (0.19) | -1.01 (0.28) | -1.60 (0.28) | -0.45 (0.28) |
| c | -1.36×10-3 (1.24×10-3) | -2.52×10-3 (2.71×10-3) | -1.69×10-3 (1.76×10-3) | -0.51×10-3 (1.76×10-3) |

Supplementary Table 3: Pearson’s correlation coefficient between spring barley grain yield and summarized monthly total rainfall (a) and mean temperature (b) at different Nitrogen levels and mineral fertilizer treatments. (* P < 0.050)

| **(a)** |  |  |  |  |  |  |  |  | |
| --- | --- | --- | --- | --- | --- | --- | --- | --- | --- |
|  | **February** | **March** | **April** | **May** | **June** | **July** | **August** | **September** | |
| **0 Kg N ha-1 PKNaMg** | -0.0933 | -0.1983 | -0.3025* | -0.4216* | 0.4226* | 0.0229 | -0.1685 | -0.0082 |  |
| **48 Kg N ha-1 PKNaMg** | -0.1084 | 0.0126 | -0.2370 | -0.1817 | 0.4052* | 0.0247 | -0.0495 | -0.0583 |  |
| **96 Kg N ha-1 PKNaMg** | -0.2718 | 0.0329 | -0.2388 | -0.0714 | 0.3585* | -0.0350 | -0.0550 | -0.1464 |  |
| **144 Kg N ha-1 PKNaMg** | -0.2052 | 0.0735 | -0.0660 | 0.0001 | 0.2818 | 0.1221 | -0.0075 | -0.1630 |  |
| **0 Kg N ha-1 P** | -0.1561 | -0.1884 | -0.1070 | -0.2812 | 0.4197* | -0.0524 | 0.0535 | 0.1167 |  |
| **48 Kg N ha-1 P** | -0.2097 | 0.2093 | -0.1013 | -0.0555 | 0.3239* | -0.0175 | 0.0476 | -0.0739 |  |
| **96 Kg N ha-1 P** | -0.1095 | 0.3237* | -0.0656 | 0.0996 | 0.0854 | -0.0476 | 0.1210 | -0.0832 |  |
| **144 Kg N ha-1 P** | -0.0671 | 0.1819 | -0.1073 | 0.0236 | 0.0484 | 0.0043 | 0.0690 | -0.048 |  |
| **0 Kg N ha-1 KNaMg** | 0.1223 | -0.2475 | -0.4154* | -0.3506* | 0.3533* | 0.0178 | -0.0840 | -0.0756 |  |
| **48 Kg N ha-1 KNaMg** | 0.1421 | -0.0443 | -0.3529* | -0.1628 | 0.2439 | -0.1256 | -0.1272 | -0.2369 |  |
| **96 Kg N ha-1 KNaMg** | 0.0870 | -0.0605 | -0.3604* | -0.1941 | 0.299 | -0.1228 | -0.1574 | -0.1922 |  |
| **144 Kg N ha-1 KNaMg** | 0.1272 | 0.0412 | -0.2806 | -0.1277 | 0.1719 | -0.1295 | -0.1242 | -0.1841 |  |
| **0 Kg N ha-1** | 0.1737 | -0.1833 | -0.2992* | -0.1699 | 0.2255 | 0.0580 | 0.1210 | -0.1350 |  |
| **48 Kg N ha-1** | 0.1341 | 0.0247 | -0.2232 | -0.1249 | 0.1835 | -0.0255 | 0.0395 | -0.2144 |  |
| **96 Kg N ha-1** | 0.1501 | -0.0171 | -0.2955 | -0.1034 | 0.1254 | -0.0224 | 0.0042 | -0.2223 |  |
| **144 Kg N ha-1** | 0.2180 | 0.0617 | -0.2026 | -0.0611 | 0.0444 | -0.0533 | 0.0909 | -0.2587 |  |
|  |  |  |  |  |  |  |  |  |  |
| **(b)** |  |  |  |  |  |  |  |  |  |
|  | **February** | **March** | **April** | **May** | **June** | **July** | **August** | **September** |  |
| **0 Kg N ha-1 PKNaMg** | -0.0301 | -0.0224 | 0.0764 | -0.0824 | -0.0275 | -0.0141 | 0.0349 | 0.0290 |  |
| **48 Kg N ha-1 PKNaMg** | -0.0647 | 0.0384 | -0.1411 | -0.0983 | -0.2448 | -0.2244 | -0.1548 | -0.0744 |  |
| **96 Kg N ha-1 PKNaMg** | -0.1944 | -0.1011 | -0.0099 | -0.1709 | -0.1567 | -0.2310 | -0.246 | 0.0366 |  |
| **144 Kg N ha-1 PKNaMg** | -0.1257 | -0.0123 | -0.0703 | 0.0346 | -0.1260 | -0.1611 | -0.1455 | -0.0024 |  |
| **0 Kg N ha-1 P** | -0.0810 | 0.1714 | 0.1888 | -0.0852 | -0.2020 | -0.0143 | -0.1824 | 0.0469 |  |
| **48 Kg N ha-1 P** | -0.2592 | -0.0051 | 0.0731 | -0.1236 | -0.2867* | -0.1903 | -0.3626* | 0.0026 |  |
| **96 Kg N ha-1 P** | -0.2439 | -0.1473 | -0.0181 | -0.1090 | -0.1828 | -0.2249 | -0.3110* | -0.0139 |  |
| **144 Kg N ha-1 P** | -0.2402 | -0.1023 | 0.1083 | -0.0833 | -0.2086 | -0.0996 | -0.1982 | -0.0343 |  |
| **0 Kg N ha-1 KNaMg** | -0.1018 | -0.0053 | 0.1300 | -0.3456* | -0.2604 | -0.2391 | 0.0210 | -0.1956 |  |
| **48 Kg N ha-1 KNaMg** | -0.2779 | -0.2324 | -0.1348 | -0.4761* | -0.4068* | -0.3766* | -0.1308 | -0.2558 |  |
| **96 Kg N ha-1 KNaMg** | -0.2853* | -0.2505 | -0.0481 | -0.4633* | -0.4528* | -0.3596* | -0.1701 | -0.2072 |  |
| **144 Kg N ha-1 KNaMg** | -0.2443 | -0.2186 | -0.2194 | -0.3366* | -0.4481* | -0.4263* | -0.2529 | -0.2034 |  |
| **0 Kg N ha-1** | -0.1076 | -0.0512 | 0.1533 | -0.3325* | -0.2738 | -0.3163* | -0.0917 | -0.2367 |  |
| **48 Kg N ha-1** | -0.2383 | -0.2840 | -0.1382 | -0.3773* | -0.4940* | -0.3331* | -0.1750 | -0.2208 |  |
| **96 Kg N ha-1** | -0.3014* | -0.2622 | -0.0721 | -0.4359* | -0.5502* | -0.2713 | -0.1750 | -0.3075* |  |
| **144 Kg N ha-1** | -0.2005 | -0.2566 | -0.1044 | -0.3204* | -0.4785* | -0.3195* | -0.2846* | -0.2811 |  |
