# Supplementary figures and images for: Investigating the effects of inter-annual weather variation (1968–2016) on the functional response of cereal grain yield to applied nitrogen, using data from the Rothamsted Long-Term Experiments
Source: Agric For Meteorol. 2020 Apr 15;284:107898. doi: 10.1016/j.agrformet.2019.107898 (PMC7079297; doi:10.1016/j.agrformet.2019.107898)

Supplementary Figure 1

(a)

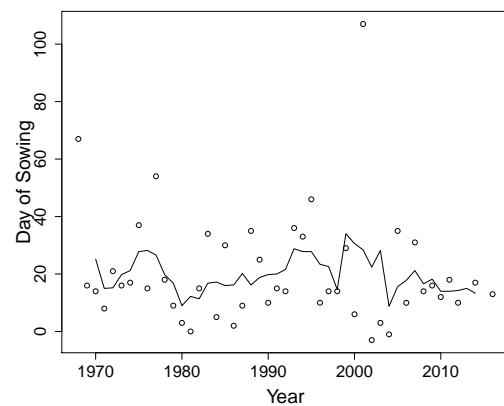

(b)

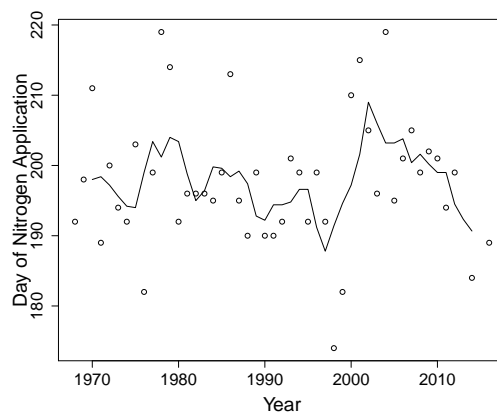

(c)

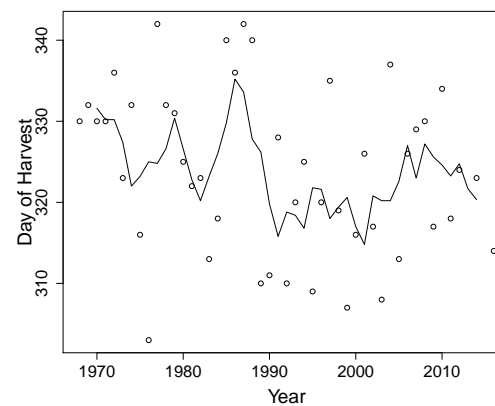

(d)

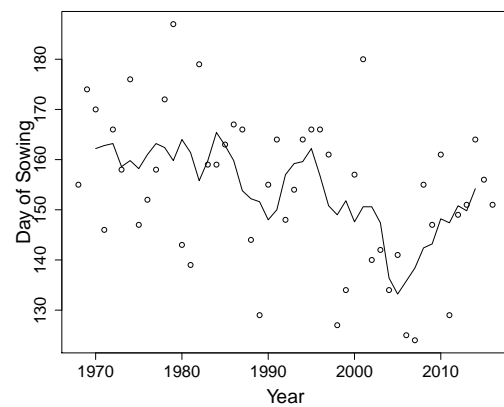

(e)

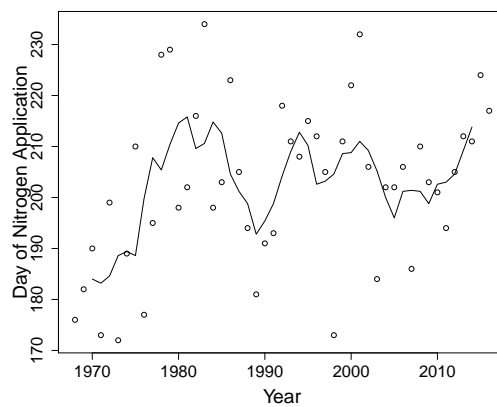

(f)

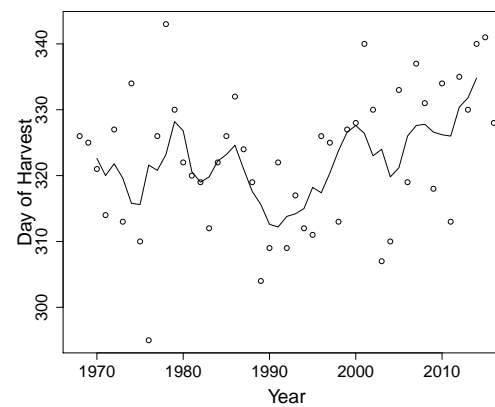

Supplement: Supplementary file 2 — Supplementary Fig. 1. The sowing (a), nitrogen application (b) and harvest dates (c) for the Broadbalk wheat experiment. The sowing (d), nitrogen application (e) and harvest dates for the Hoosfield spring barley experiment. Data includes harvest seasons 1968 to 2016 (2013 and 2015 were excluded from Broadbalk due to late sowing). The solid line represents a five-year rolling mean, with dates expressed relative to the 1st of October for each year. [file mmc2.pdf]

Supplementary Figure 2

(a)

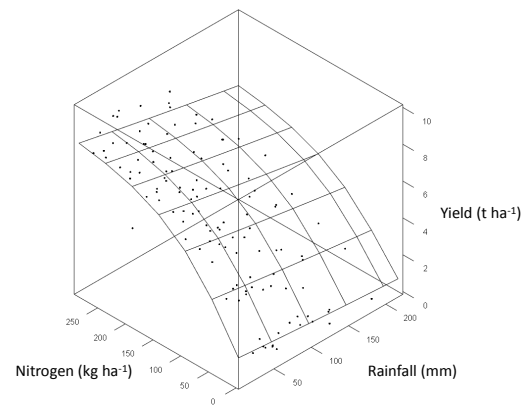

(b)

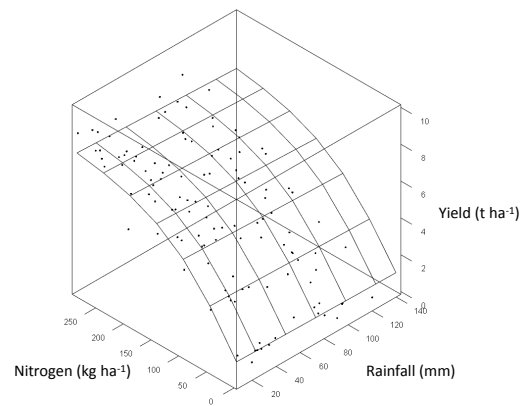

(c)

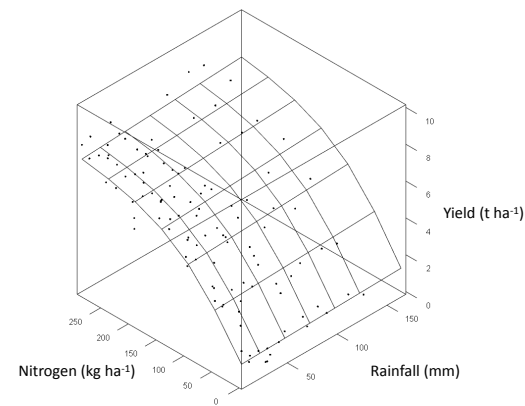

Supplement: Supplementary file 3 — Supplementary Figure 2. Response surface (Equation (4), Table 3) of the effect of applied nitrogen (kg N ha−1) on winter wheat grain yield (•, t ha−1 at 85% dry matter) from Section 1 (all with PKNaMg) of the Broadbalk Long Term Experiment, adjusted for cultivar Hereward, as affected by: (a) Total October rainfall; (b) Total February rainfall; (c) Total June rainfall. [file mmc3.pdf]

Supplementary Figure 3

(a)

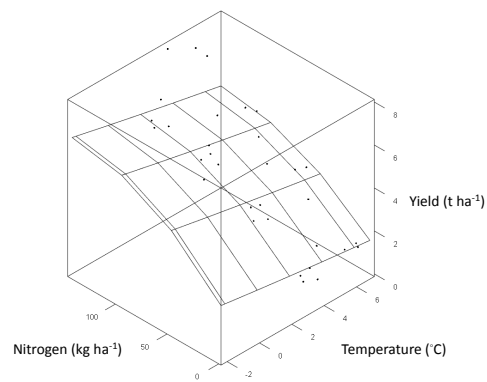

(b)

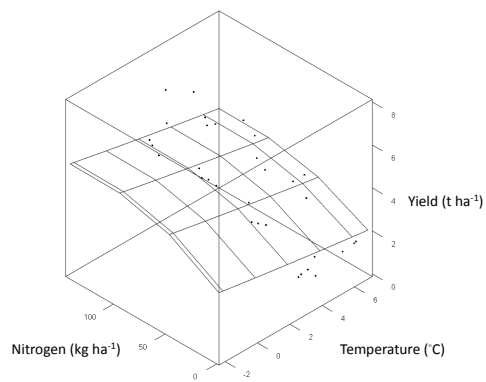

(c)

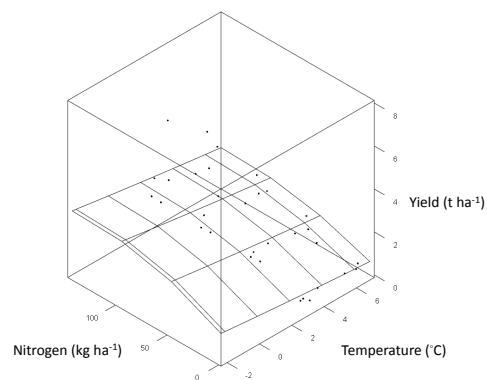

(d)

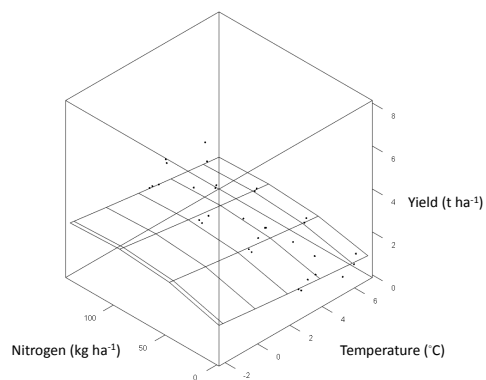

Supplement: Supplementary file 4 — Supplementary Figure 3. Response surface (Equation (5), Table 4) for the effect of applied nitrogen (kg N ha−1) on spring barley grain yield (•, t ha−1 at 85% dry matter), adjusted for cultivar Tipple, as affected by mean February temperature for mineral fertilizer treatments (a) PKNaMg, (b) P, (c) KNaMg, or (d) Nil. [file mmc4.pdf]

Supplementary Figure 4  
(a)

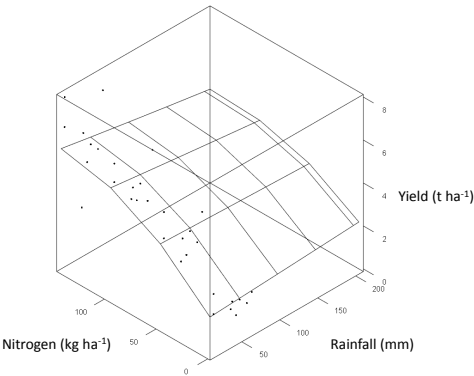

(b)

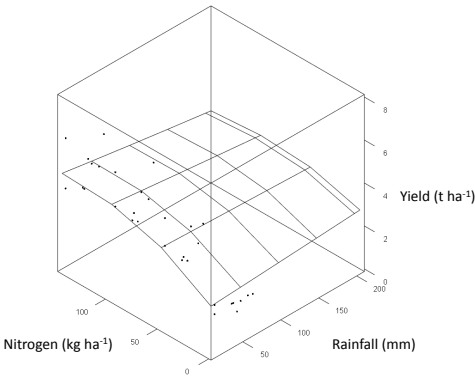

(c)

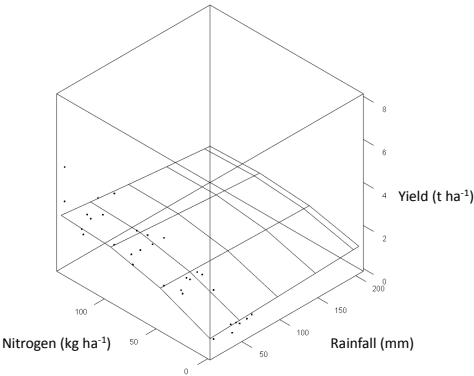

(d)

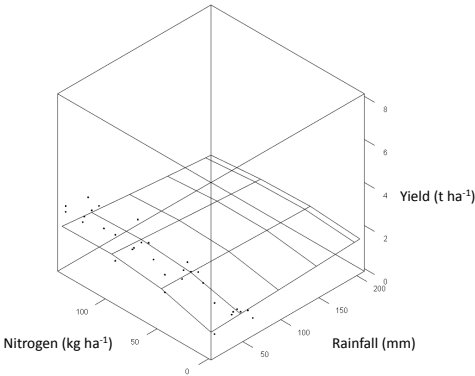

Supplement: Supplementary file 5 — Supplementary Figure 4. Response surface (Equation (5), Table 4) for the effect of applied nitrogen (kg N ha−1) on spring barley grain yield (•, t ha−1 at 85% dry matter), adjusted for cultivar Tipple, as affected by September rainfall for mineral fertilizer treatments (a) PKNaMg, (b) P, (c) KNaMg, or (d) Nil. [file mmc5.pdf]
